# Composite Assessment of Menstrual Irregularity and Its Determinants Among Medical and Non‐Medical Female Undergraduates in Chittagong, Bangladesh: A Cross‐Sectional Study

**DOI:** 10.1002/hsr2.72448

**Published:** 2026-04-27

**Authors:** Md. Mayin Uddin Hasan, Brinta Bhowmik, Mohammad Injamul Hoq, Mohammed Aktar Sayeed, Md. Zakir Sultan

**Affiliations:** ^1^ Department of Pharmacy International Islamic University Chittagong Chattogram Kumira Bangladesh; ^2^ Drug Insides and Disease Epidemiology‐DIDE Chattogram Khulshi Bangladesh; ^3^ Department of Public Health University of Creative Technology Chittagong Chattogram Chattogram Bangladesh; ^4^ Center for Advanced Research in Sciences University of Dhaka Dhaka Dhaka Bangladesh

**Keywords:** Bangladesh, menstrual health, menstrual irregularities, perceived stress, undergraduate students

## Abstract

**Background and Aim:**

Menstrual irregularities affect women's physiological, psychological, and reproductive health, yet remain understudied among university students in Bangladesh. This study aimed to assess menstrual characteristics using a composite menstrual irregularity (CMI) score and identified key determinants among female undergraduates in Chittagong.

**Methods:**

An institution‐based cross‐sectional study was conducted among 469 female undergraduates from three universities in Chittagong, Bangladesh (September 2024–May 2025). The CMI score and Perceived Stress Scale (PSS‐10) were used for assessing menstrual irregularity and stress level respectively. Multivariable logistic regression models have been applied; results are shown as adjusted odds ratios (AORs) with 95% confidence intervals (CIs).

**Results:**

Of 469 participants (240 non‐medical, 229 medical; median age 22 years [IQR 21–23]), 81.2% met CMI‐1 (mild irregularity), 45.5% met CMI‐2 (moderate irregularity), and 16.9% met CMI‐3 (severe irregularity). Metrorrhagia was the strongest predictor across all thresholds (CMI‐1: AOR 4.37 [1.26–27.65]; CMI‐2: AOR 3.12 [1.53–6.71]; CMI‐3: AOR 3.27 [1.53–6.83]; all *p* < 0.05). Middle‐range income (20,000–40,000 BDT) elevated CMI‐1 odds (AOR 5.52 [1.78–17.88]; *p* = 0.003). In stratified analyzes, late menarche predicted irregularity exclusively among non‐medical students (CMI‐1: AOR 2.71 [1.22–6.50]; CMI‐2: AOR 1.93 [1.06–3.57]; CMI‐3: AOR 2.37 [1.08–5.31]; all *p* < 0.05); married status was protective against CMI‐2 in non‐medical students (AOR 0.24 [0.08–0.65]; *p* = 0.006); and hostel residence increased CMI‐2 odds in medical students (AOR 2.73 [1.17–6.72]; *p* = 0.023). High perceived stress was not independently associated with any threshold. The Internet and mass media were the primary sources of menstrual knowledge (52.5%).

**Conclusions:**

Menstrual irregularity is prevalent and multidimensionally patterned among female undergraduates in Bangladesh. Perceived stress, though elevated among non‐medical students, was not independently associated with irregularity in adjusted models. Metrorrhagia, income, late menarche, and living arrangements are key modifiable factors. These findings support targeted university health interventions and evidence‐based menstrual health policies in Bangladesh.

## 1 Introduction

Menstruation is a regular physiological occurrence in women of reproductive age, beginning with menarche in adolescence and continuing until menopause. It characterizes the periodic loss of the uterine lining and hormonal changes. Menstrual health refers to a person's physical, emotional, and social well‐being throughout their menstrual cycle. Although a universal biological process, menstrual health remains an overlooked public health concern, especially in low‐ and middle‐income countries (LMICs), where sociocultural stigma, limited health literacy, and restricted access to reproductive healthcare combine to negatively affect women's menstrual experiences and quality of life [1, 2]. It may be affected by a range of environmental conditions, including age, weight, physical activity, and mental health problems such as anxiety, stress, and depression [3].

Globally, the prevalence of menstrual irregularity among women of reproductive age is estimated to range from 5% to 35.6%, with notably higher rates — reaching up to 90.4% for any menstrual disorder—reported among university student populations, who form a particularly vulnerable group due to their exposure to combined academic, nutritional, and psychosocial stressors [4, 5, 6]. Previous research has demonstrated a correlation between irregular menstruation and socioeconomic factors, including family income and educational level [7]. Major contributing factors to menstrual irregularity include nutritional status, anemia, and genetic factors [8]. Studies have also shown that obesity, stress, and smoking are correlated with irregular menstruation and premature menopause [9].

Genetic predisposition and lifestyle factors, including eating choices, physical activity, and sleeping habits, have been identified as influencing menstrual irregularities [10, 11, 12, 13]. Women who experience heavy periods tend to skip school and work activities as well as social engagements in comparison to women with light or moderate periods [14]. During menstruation, women experience poor sleep quality, which might affect their academic responsibilities and negatively impact productivity, self‐determination, and academic motivation [15, 16]. Underweight women, insomnia, excessive alcohol consumption, stress, anemia, or hereditary factors are vital contributors to menstrual irregularity [8, 13, 17].

University students worldwide experience disproportionately high perceived stress levels compared to same‐age non‐student groups, caused by academic pressure, financial insecurity, exam demands, social adjustment, and, among residential students, separation from family support networks [18, 19]. A systematic review of Japanese college students showed that higher PSS scores were significantly linked to irregular menstrual cycles and more severe premenstrual symptoms [20]. Similarly, a study involving Saudi health sciences students revealed that 62.7% of those experiencing irregular cycles reported high perceived stress, whereas only 37.8% of students with regular cycles [4]. Overall, this body of evidence emphasizes that perceived stress is a plausible factor of menstrual disruption in academic populations.

Menstrual irregularities can harm students' academic and social well‐being. Studies have demonstrated that students who are absent from classes experience poorer academic performance and face multiple adverse effects due to issues related to menstruation [13, 21]. Medical students face significant challenges in maintaining their menstrual health due to the demanding nature of their academic and clinical environments. Research highlights the high prevalence of menstrual disorders in this population. An Indian study reported that the Prevalence of premenstrual syndrome, dysmenorrhea, and amenorrhea are the most common problems reported [22]. These cases result in college absenteeism, and 45.38% of students miss classes due to menstrual problems [23].

Menstrual health in Bangladesh faces significant socio‐cultural and systemic challenges. Widespread stigma, micronutrient deficiencies, and premenstrual symptoms further compound these issues [24, 25]. Several studies have explored menstrual health management, cultural taboos, and health implications associated with menstrual disorders in Bangladesh [26, 27, 28, 29]. Bangladesh's unique epidemiological context is characterized by a high burden of anemia (estimated at 41.5% among adolescent girls), Widespread food insecurity, pervasive menstrual stigma that inhibits open discussion and healthcare access, and a reproductive health system that has traditionally focused on maternal and child health rather than menstrual health services [30, 31, 32]. In urban centers such as Chittagong, Bangladesh's second‐largest city and a key commercial hub, rapid urbanization has introduced several stressors. These include a shift to processed foods in diets, more sedentary lifestyles, and competitive academic environments, all of which increase the menstrual health risks for young women.

Notably, no previous research in Bangladesh has simultaneously examined menstrual health outcomes and perceived stress among both medical and non‐medical university students, a comparison essential for understanding how academic environments affect menstrual health and for creating tailored, student‐focused health interventions. This study aims to assess menstrual characteristics and the multidimensional menstrual irregularities using a Composite Menstrual Irregularity (CMI) framework, examine perceived stress levels, and identify factors associated with menstrual irregularity among medical and non‐medical female undergraduate students in Chittagong, Bangladesh.

## 2 Methodology

### 2.1 Study Design

An institution‐based, cross‐sectional study was conducted among female undergraduate students from three universities in Chittagong City, Bangladesh, from September 2024 to May 2025. The universities included Chittagong Medical University (CMU), the International Islamic University of Chittagong (IIUC), and the University of Creative Technology Chittagong (UCTC).

### 2.2 Sampling Technique

Three institutions, including Chittagong Medical University (CMU), International Islamic University Chittagong (IIUC), and the University of Creative Technology Chittagong (UCTC), were purposively selected based on their enrollment of both medical and non‐medical students, thereby ensuring a diverse sample amenable to comparative analysis. Within these institutions, participants were selected using a stratified random sampling approach.

The student population was first stratified into two broad groups: medical and non‐medical. The medical stratum comprised students enrolled in Bachelor of Medicine and Bachelor of Surgery (MBBS), Bachelor of Dental Surgery (BDS), and Bachelor of Pharmacy (B. Pharm) programs at CMU and IIUC. The non‐medical stratum included students from the departments of Computer Science and Engineering (CSE), Electrical and Electronic Engineering (EEE), English Language and Literature (ELL), Bachelor of Business Administration (BBA), and Economics and Banking (EB) at IIUC and UCTC.

**Figure 1:**
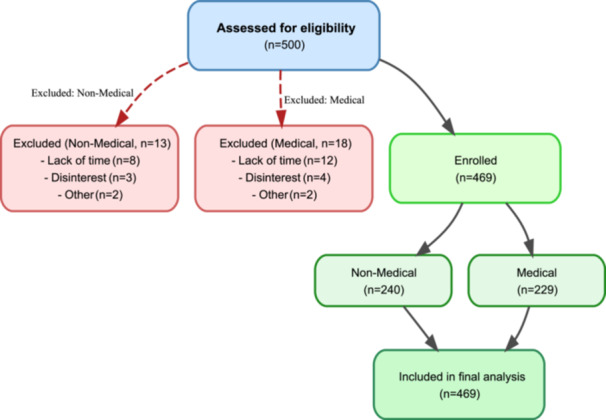
CONSORT diagram of participant enrollment and retention.

To determine the method of participant assignment to the various strata, a proportional allocation approach was employed, allocating the sample size to each stratum based on its proportion of the total population across the three institutions. This was calculated as:

$$n_{h}=\left(\frac{N_{h}}{N}\right)\times n,$$

where $n_{h}$ denotes the sample size for stratum h, $N_{h}$ is the population size of that stratum, N is the total population across all three institutions, and n is the overall target sample size. Within each stratum, simple random sampling was subsequently applied, ensuring that every eligible student had an equal probability of selection. As detailed in the CONSORT diagram (Figure 1), this procedure yielded a final analyzed sample of 229 medical and 240 non‐medical students. The approach reduced selection bias while ensuring a balanced representation of both cohorts for comparison.

### 2.3 Inclusion and Exclusion Criteria

Medical and non‐medical students aged 18 years or above from selected universities were included in the study. The exclusion criteria were anyone below the age of 18 with a chronic illness or pregnancy. Additionally, this applies to individuals with any medical or mental conditions, cognitive impairments, and those who are unwilling to participate in the study.

### 2.4 Sample Size Calculation

The required sample size was estimated using the Cochran formula:

$$n=\frac{Z^{2}\mathrm{pq}}{d^{2}},$$

where *Z* = 1.96 (95% confidence level), *p* = 0.50 (assumed proportion, maximizing sample size in the absence of prior local data), q = 1 − *p* = 0.50, and d = 0.05 (5% margin of error), yielding a base estimate of *n* = 384. This was adjusted upward by a stratified design effect of 1.2 (reflecting modest between‐stratum variance in a two‐group stratified design), producing an adjusted estimate of 461. To account for an anticipated non‐response rate of 8%, 500 participants were approached. Of these, 31 were excluded due to lack of time or unwillingness to participate (13 non‐medical, 18 medical), yielding a final analyzed sample of 469 (response rate: 93.8%) (Figure 1).

### 2.5 Data Collection Tools and Procedure

The researchers used a validated questionnaire to collect data from the individuals. The questionnaire consisted of closed‐ended, validated questions from previously published studies [13, 33, 34, 35]. Data collection was conducted using paper‐based surveys distributed in person to participants. The samples were selected randomly from different departments across medical group students pursuing degrees such as Bachelor of Medicine, Bachelor of Surgery (MBBS), Bachelor of Dental Surgery (BDS), and Bachelor of Pharmacy (B. Pharm) at CMU and IIUC. The non‐medical group comprised students from various departments, including Computer Science and Engineering (CSE), Electrical and Electronic Engineering (EEE), English Language and Literature (ELL), Bachelor of Business Administration (BBA), and Economics and Banking (EB), from IIUC and UCTC. Before completing the survey, participants received a written consent form explaining the study objectives, voluntary participation, confidentiality, and data protection measures. Only those who provided informed consent proceeded to complete the survey.

#### 2.5.1 Variables Used

- **Demographic characteristics.**

Data collection included information from various demographic variables. Enrolled Education Year program participants were categorized into First, Second, Third‐, and Fourth‐Year divisions. The survey included two additional variables: Marital Status (Single or Married) and Family Type (Nuclear or Joint). Participants also disclosed their living arrangements (Living with Family or Living in a Hostel) and their previous living location (Urban or Rural). The study categorized participants' Religions into Muslim and Hindu groups and family income into four categories: < 20,000 BDT, 20,000–40,000 BDT, 40,001–60,000 BDT, and > 60,000 BDT.

- **Composite menstrual irregularity (CMI) score:**
  Menstrual irregularity was operationalized as a multidimensional, self‐reported construct using the composite menstrual irregularity (CMI) score, an instrument developed for the present study that integrates four clinically validated parameters of menstrual function into a single summary measure. This approach was adopted because a growing body of epidemiological evidence indicates that comprehensive assessment of menstrual dysfunction requires the simultaneous evaluation of cycle length, flow duration, flow volume, and subjective cycle regularity, rather than reliance on any single indicator, which is susceptible to both classification error and ceiling effects [36, 37, 38, 39].
  Each parameter was operationalized using internationally accepted clinical reference standards. Menstrual cycle length was classified as abnormal if reported as fewer than 21 days or greater than 35 days, in accordance with criteria established by the American College of Obstetricians and Gynecologists and the National Health Service. Flow duration was classified as abnormal if reported as fewer than 3 days or greater than 7 days [40, 41]. Flow volume was classified as abnormal if a participant reported using fewer than 4 or more than 8 menstrual pads per day, consistent with the FIGO criteria for the quantification of heavy menstrual bleeding [42]. Subjective menstrual regularity was classified as abnormal if the participant reported irregular cycles, in accordance with the standardized terminological framework established by Fraser et al. (2007), which defines cycle irregularity as clinically meaningful variation in the onset of menstrual bleeding that falls outside a woman's established pattern [43].
  Each parameter was scored dichotomously (0 = within normal limits; 1 = outside normal limits), yielding a total CMI score ranging from 0 to 4, with higher scores reflecting a greater number of concurrently abnormal menstrual dimensions. To minimize threshold‐selectivity bias—a well‐recognized limitation of studies that define menstrual irregularity using a single binary cut‐point—three pre‐specified severity thresholds were applied to the CMI score [44]: CMI‐1 (score ≥ 1) defined the presence of *any* menstrual abnormality across the four dimensions or mild irregularity, thereby maximizing case detection sensitivity; CMI‐2 (score ≥ 2) indicated *moderate* multi‐dimensional irregularity, providing a balance between sensitivity and specificity; and CMI‐3 (score ≥ 3) captured *severe*, multi‐feature menstrual irregularity characterized by high specificity for clinically significant dysfunction. The use of multiple graded outcome thresholds to characterize both the prevalence and severity of menstrual irregularity in epidemiological research has been applied in prior studies of menstrual health in academic and occupational populations [19, 20]. This self‐reported, cross‐sectional approach enabled systematic characterization of menstrual irregularity patterns across the study population while remaining aligned with internationally endorsed clinical definitions.
- **Perceived stress scale (PSS):**

We included the perceived stress scale (PSS) in the questionnaire to facilitate the assessment of their stress levels. We used the PSS, which assesses cognitive and emotional experiences over the previous month, to evaluate their stress levels. The scale uses a 5‐point Likert scale ranging from 0 to 4. The total number of replies indicates the overall score. The PSS‐10 yields scores ranging from zero to forty (0–40). Participants with ≤ 20 and > 20 PSS values represented the low‐stress and high‐stress groups, respectively [13].

### 2.6 Statistical Analysis

Sociodemographic characteristics, menstrual patterns, and perceived stress levels were summarized using descriptive statistics. The normality of continuous variables, including age and PSS scores, was evaluated using the Shapiro‐Wilk test; normally distributed data are reported as mean (SD), while non‐normally distributed data are presented as median (IQR). Bivariate associations for categorical variables were assessed using the chi‐square test, whereas continuous exposures were compared using Mann‐Whitney *U* tests. To identify independent predictors of menstrual irregularity, binary logistic regression models were constructed for each pre‐specified CMI outcome threshold to minimize threshold selectivity bias. Results are reported as adjusted odds ratios (AOR) with 95% CIs. To account for suspected effect modification by academic background, separate multivariable models were fitted for medical and non‐medical students, following the stratified approach [44]. The discriminatory ability of these models was assessed using the area under the receiver operating characteristic curve (AUC‐ROC). AUC 95% CIs were calculated with the DeLong nonparametric method to account for the correlated nature of sensitivity‐specificity pairs. Multicollinearity was assessed using the Variance Inflation Factor (VIF) for all independent predictors. A VIF threshold of < 5.0 was used to ensure stable regression coefficients and the absence of redundant variables. Statistical significance was defined as *p* < 0.05 (two‐tailed), with exact p‐values reported; *p* < 0.10 indicated marginal significance or trends. Statistical analyzes were performed using Stata (version 18; StataCorp, College Station, TX, USA) and R version 4.5.1.

### 2.7 Ethics issues

The Institutional Review Board (IRB) of the University of Creative Technology, Chittagong, Bangladesh, Ref: IRB/UCTC/2024/101, approved the study. Participants' data were kept confidential, and participants were included in the study after providing informed written consent. Participants' confidentiality was maintained by anonymizing responses, securely storing data, and restricting access to authorized researchers only.

## 3 Results

### 3.1 Participant Characteristics

Of 469 participants, 240 were non‐medical students, and 229 were medical students. The median age was 22 years (IQR 21–23) in both groups. Several sociodemographic characteristics differed significantly between the groups. Married status was more common among non‐medical students (12.9% vs 4.4%; *p* = 0.002), as was living in a joint family (28.7% vs 18.8%; *p* = 0.015). Non‐medical students were more likely to have lived in a rural area before university (20.0% vs 10.9%; *p* = 0.010), and family income distribution differed significantly between groups (*p* = 0.034), with higher‐income households more common among medical students. Religion and current residence did not differ significantly. Self‐reported menstrual irregularity was significantly more common among medical students than non‐medical students (24.5% vs. 16.7%; *p* = 0.048). Cycle length distribution also differed significantly (*p* = 0.041): short cycles (< 21 days) were more prevalent in non‐medical students (13.4% vs 6.6%), while long cycles (> 35 days) were more common in medical students (14.0% vs 11.3%) (Table 1).

**Table 1:** Sociodemographic, menstrual, and psychological characteristics of female undergraduate students by academic background (*N* = 469).

Characteristic	Overall (*N* = 469)	Non‐Medical (*n* = 240)	Medical (*n* = 229)	*p*‐value
Sociodemographic				
Age (years), median [IQR]	22.0 (21–23)	22.0 (21–23)	22.0 (21–23)	0.071#
University				< 0.001*,$
IIUC	287 (61.2%)	197 (82.1%)	90 (39.3%)	
UCTC	43 (9.2%)	43 (17.9%)	0 (0.0%)	
CMU	139 (29.6%)	0 (0.0%)	139 (60.7%)	
Academic year				< 0.001*,$
1st year	128 (27.3%)	66 (27.5%)	62 (27.1%)	
2nd year	105 (22.4%)	71 (29.6%)	34 (14.8%)	
3rd year	103 (22.0%)	42 (17.5%)	61 (26.6%)	
4th year	133 (28.4%)	61 (25.4%)	72 (31.4%)	
Marital status				0.002*,$
Single	428 (91.3%)	209 (87.1%)	219 (95.6%)	
Married	41 (8.7%)	31 (12.9%)	10 (4.4%)	
Family type				0.015*,$
Nuclear	357 (76.1%)	171 (71.2%)	186 (81.2%)	
Joint	112 (23.9%)	69 (28.7%)	43 (18.8%)	
Current residence				0.932$
With family	410 (87.4%)	209 (87.1%)	201 (87.8%)	
Hostel	59 (12.6%)	31 (12.9%)	28 (12.2%)	
Pre‐admission residence				0.010*,$
Urban	396 (84.4%)	192 (80.0%)	204 (89.1%)	
Rural	73 (15.6%)	48 (20.0%)	25 (10.9%)	
Religion				0.912$
Muslim	448 (95.5%)	230 (95.8%)	218 (95.2%)	
Hindu	21 (4.5%)	10 (4.2%)	11 (4.8%)	
Family income (BDT)				0.034*,$
< 20,000	32 (6.8%)	22 (9.2%)	10 (4.4%)	
20,000–40,000	98 (20.9%)	58 (24.2%)	40 (17.5%)	
40,001–60,000	187 (39.9%)	89 (37.1%)	98 (42.8%)	
> 60,000	152 (32.4%)	71 (29.6%)	81 (35.4%)	
Menstrual characteristics				
Age at menarche				0.051$
9–10 years	28 (6.0%)	14 (5.8%)	14 (6.1%)	
11–13 years	291 (62.0%)	137 (57.1%)	154 (67.2%)	
14–16 years	150 (32.0%)	89 (37.1%)	61 (26.6%)	
Menstrual irregularity				0.048*,$
No	373 (79.5%)	200 (83.3%)	173 (75.5%)	
Yes	96 (20.5%)	40 (16.7%)	56 (24.5%)	
Cycle length				0.041*,$
< 21 days	47 (10.0%)	32 (13.4%)	15 (6.6%)	
21–35 days	362 (77.4%)	180 (75.3%)	182 (79.5%)	
> 35 days	59 (12.6%)	27 (11.3%)	32 (14.0%)	
Duration of flow				0.089$
< 3 days	53 (11.3%)	34 (14.2%)	19 (8.3%)	
3–7 days	384 (82.1%)	192 (80.3%)	192 (83.8%)	
> 8 days	31 (6.6%)	13 (5.4%)	18 (7.9%)	
Amount of flow				0.353$
Little (< 4 pads)	250 (53.4%)	120 (50.2%)	130 (56.8%)	
Moderate (5–7 pads)	197 (42.1%)	107 (44.8%)	90 (39.3%)	
Heavy (≥ 8 pads)	21 (4.5%)	12 (5.0%)	9 (3.9%)	
Menstrual pathologies				
Dysmenorrhea				0.808$
No	229 (48.8%)	119 (49.6%)	110 (48.0%)	
Yes	240 (51.2%)	121 (50.4%)	119 (52.0%)	
Menorrhagia				0.050$
No	377 (80.4%)	184 (76.7%)	193 (84.3%)	
Yes	92 (19.6%)	56 (23.3%)	36 (15.7%)	
Metrorrhagia				0.992$
No	429 (91.5%)	219 (91.2%)	210 (91.7%)	
Yes	40 (8.5%)	21 (8.8%)	19 (8.3%)	
History of menstrual pathologies				
Ever had amenorrhea				0.576$
No	257 (54.8%)	128 (53.3%)	129 (56.3%)	
Yes	212 (45.2%)	112 (46.7%)	100 (43.7%)	
Duration of missed period‡				0.905$
< 3 months	140 (66.0%)	68 (78.2%)	72 (77.4%)	
≥ 3 months	40 (18.9%)	19 (21.8%)	21 (22.6%)	
Not sure	32 (15.1%)	—	—	
Psychological Stress				
PSS score, median (IQR)	23.0 [20–25]	23.0 [21–25]	22.0 (19–25)	0.024*,$
Stress category				0.051$
Low stress	135 (28.8%)	59 (24.6%)	76 (33.2%)	
High stress	334 (71.2%)	181 (75.4%)	153 (66.8%)	
Composite outcomes (*N* = 468)				
CMI‐1: MI ≥ 1				0.917$
Regular	88 (18.8%)	44 (18.4%)	44 (19.2%)	
Irregular	380 (81.2%)	195 (81.6%)	185 (80.8%)	
CMI‐2: MI ≥ 2				0.489$
Regular	255 (54.5%)	126 (52.7%)	129 (56.3%)	
Irregular	213 (45.5%)	113 (47.3%)	100 (43.7%)	
CMI‐3: MI ≥ 3				0.969$
Regular	389 (83.1%)	198 (82.8%)	191 (83.4%)	
Irregular	79 (16.9%)	41 (17.2%)	38 (16.6%)	

Significance level:

^#^ Mann‐Whitney *U* test.

^$^ Chi‐square test.

^‡^ Among respondents with ever had amenorrhea (*n* = 212).

* *p* < 0.05.

Non‐medical students had a significantly higher median PSS score than medical students (23.0 [IQR 21–25] vs 22.0 [IQR 19–25]; *p* = 0.024). The difference in high‐stress prevalence (75.4% among non‐medical students vs. 66.8% among medical students) was not statistically significant (*p* = 0.051). Using the CMI framework, 81.2% of participants met CMI‐1 criteria (mild irregularity), 45.5% met CMI‐2 criteria (moderate irregularity), and 16.9% met CMI‐3 criteria (severe multi‐feature irregularity). Notably, these distributions did not differ significantly between medical and non‐medical students at any threshold (Table 1).

### 3.2 Perceived Stress Scale (PSS‐10)

Analysis of individual PSS‐10 items revealed significant differences based on academic background and primary outcome status. Specifically, participants with a non‐medical background reported significantly higher stress levels related to being upset by unexpected events (*p* = 0.029) and lower confidence in handling personal problems (*p* = 0.018) compared to their medical counterparts. Furthermore, those meeting the primary outcome (MI) criteria demonstrated significantly higher scores for being upset by unexpected events than the regular group (*p* = 0.012). No other item‐level comparisons reached statistical significance (see Supporting Information Table S1 for complete item‐level data).

### 3.3 Factors Associated With Composite Menstrual Irregularity

The association between the associated factor and composite menstrual irregularity is shown in Table 2. Metrorrhagia was the strongest and most consistent independent predictor across all three CMI thresholds. Compared to students without metrorrhagia, those with metrorrhagia were 4.37 times more likely to meet the CMI‐1 criteria (AOR 4.37, 95% CI 1.26–27.65; *p* = 0.049), 3.12 times more likely to meet the CMI‐2 criteria (AOR 3.12, 95% CI 1.53–6.71; *p* = 0.002), and 3.27 times more likely to meet the CMI‐3 criteria (AOR 3.27, 95% CI 1.53–6.83; *p* = 0.002).

**Table 2:** Associated factors influencing composite menstrual irregularity at three severity thresholds among female undergraduate students (*N* = 468).

Predictor variable	Model 1				Model 2				Model 3			
	CMI‐1: Composite MI score ≥ 1				CMI‐2: Composite MI score ≥ 2				CMI‐3: Composite MI score ≥ 3			
	COR (95% CI)	*p*‐value	AOR (95% CI)	*p*‐value	COR (95% CI)	*p*‐value	AOR (95% CI)	*p*‐value	COR (95% CI)	*p*‐value	AOR (95% CI)	*p*‐value
Academic Background (ref: non‐medical)												
Medical	0.95 (0.60–1.51)	0.824	1.00 (0.60–1.68)	0.987	0.86 (0.60–1.24)	0.433	0.89 (0.60–1.34)	0.582	0.96 (0.59–1.56)	0.871	0.97 (0.56–1.66)	0.902
Academic year (ref: 1st year)												
2nd year	1.31 (0.68–2.56)	0.431	1.39 (0.69–2.85)	0.359	0.83 (0.49–1.39)	0.471	0.85 (0.49–1.49)	0.575	0.69 (0.31–1.47)	0.344	0.71 (0.31–1.57)	0.402
3rd year	1.19 (0.62–2.32)	0.596	1.33 (0.59–3.02)	0.486	1.08 (0.64–1.82)	0.774	0.99 (0.52–1.89)	0.972	0.91 (0.43–1.88)	0.804	0.85 (0.34–2.07)	0.718
4th year	1.23 (0.66–2.28)	0.514	1.34 (0.55–3.25)	0.523	1.07 (0.66–1.75)	0.784	0.78 (0.38–1.57)	0.485	1.70 (0.92–3.20)	0.096†	1.22 (0.49–3.10)	0.676
Marital status (ref: single)												
Married	0.69 (0.34–1.54)	0.340	0.54 (0.23–1.34)	0.169	0.75 (0.38–1.43)	0.384	0.53 (0.25–1.09)	0.090†	1.22 (0.50–2.62)	0.638	0.80 (0.30–1.93)	0.632
Family type (ref: nuclear)												
Joint	1.16 (0.67–2.08)	0.603	1.28 (0.72–2.37)	0.418	1.07 (0.70–1.64)	0.747	1.09 (0.69–1.72)	0.712	0.79 (0.42–1.40)	0.428	0.80 (0.41–1.47)	0.480
Residence (ref: with family)												
Hostel	2.17 (0.97–5.79)	0.085†	2.09 (0.88–5.81)	0.121	1.33 (0.77–2.31)	0.311	1.22 (0.67–2.20)	0.514	1.03 (0.47–2.06)	0.937	0.94 (0.40–2.01)	0.876
Pre‐admission (ref: urban)												
Rural	0.95 (0.52–1.86)	0.880	0.77 (0.38–1.61)	0.471	1.24 (0.75–2.05)	0.406	1.09 (0.63–1.89)	0.759	1.10 (0.55–2.07)	0.772	1.09 (0.51–2.19)	0.819
Religion (ref: Muslim)												
Hindu	1.41 (0.46–6.11)	0.589	0.98 (0.29–4.52)	0.979	0.58 (0.22–1.43)	0.256	0.46 (0.16–1.21)	0.125	0.51 (0.08–1.79)	0.366	0.44 (0.07–1.71)	0.304
Income (ref: < 20,000 BDT)												
20,000–40,000 BDT	5.03 (1.70–15.50)	0.004**	5.52 (1.78–17.88)	0.003**	1.31 (0.59–2.93)	0.509	1.41 (0.61–3.27)	0.420	0.68 (0.27–1.84)	0.432	0.64 (0.24–1.83)	0.388
40,001–60,000 BDT	1.31 (0.54–2.96)	0.529	1.47 (0.57–3.53)	0.406	0.67 (0.31–1.43)	0.296	0.71 (0.32–1.58)	0.400	0.60 (0.25–1.53)	0.254	0.62 (0.25–1.69)	0.329
> 60,000 BDT	1.66 (0.67–3.87)	0.254	1.67 (0.63–4.20)	0.281	0.79 (0.37–1.70)	0.541	0.75 (0.34–1.70)	0.493	0.51 (0.21–1.33)	0.148	0.44 (0.17–1.23)	0.102
Age (years, continuous)	0.99 (0.84–1.17)	0.902	0.99 (0.76–1.29)	0.937	1.09 (0.96–1.24)	0.196	1.18 (0.95–1.45)	0.131	1.23 (1.04–1.48)	0.019*	1.18 (0.89–1.57)	0.240
Menarche (ref: 11–13 years)												
9–10 years	1.17 (0.46–3.60)	0.765	1.31 (0.48–4.21)	0.619	2.01 (0.91–4.61)	0.087†	2.20 (0.95–5.24)	0.068†	1.96 (0.73–4.73)	0.150	1.77 (0.62–4.54)	0.255
14–16 years	1.54 (0.92–2.68)	0.111†	1.65 (0.95–2.96)	0.083†	1.39 (0.93–2.06)	0.107†	1.45 (0.96–2.22)	0.081†	1.29 (0.76–2.16)	0.341	1.56 (0.88–2.74)	0.121
Stress (ref: Low)												
High	1.11 (0.66–1.83)	0.673	1.04 (0.60–1.78)	0.886	1.21 (0.81–1.81)	0.363	1.24 (0.80–1.92)	0.337	1.06 (0.63–1.85)	0.830	1.17 (0.66–2.15)	0.604
Dysmenorrhea (ref: No)												
Yes	1.01 (0.63–1.60)	0.976	0.87 (0.52–1.42)	0.570	1.10 (0.76–1.58)	0.607	0.99 (0.67–1.47)	0.975	1.40 (0.86–2.30)	0.177	1.23 (0.72–2.10)	0.451
Menorrhagia (ref: No)												
Yes	1.51 (0.82–2.98)	0.203	1.59 (0.82–3.27)	0.184	1.19 (0.75–1.87)	0.465	1.14 (0.69–1.88)	0.620	1.76 (0.99–3.03)	0.046*	1.63 (0.87–2.99)	0.122
Metrorrhagia (ref: No)												
Yes	4.78 (1.43–29.73)	0.033*	4.37 (1.26–27.65)	0.049*	3.06 (1.55–6.41)	0.002**	3.12 (1.53–6.71)	0.002**	3.41 (1.68–6.76)	< 0.001***	3.27 (1.53–6.83)	0.002**

*Note:* Ref: Non‐medical, 1st year, single, nuclear, with family, Urban, Muslim, < 20,000 BDT, 11–13 years menarche, low stress, no for all pathologies. One participant was excluded from regression analyzes due to missing data, yielding an analytic sample of *N* = 468.

Abbreviations: AOR, adjusted odds ratio; COR, crude odds ratio.

*** *p* < 0.001

** *p* < 0.01

* *p* < 0.05.

^†^ *p* < 0.10.

Regarding income, students in the 20,000–40,000 BDT range were 5.52 times more likely to meet CMI‐1 criteria compared to the lowest income group (< 20,000 BDT) (AOR 5.52, 95% CI 1.78–17.88; *p* = 0.003). This income effect was not maintained at CMI‐2 or CMI‐3. Late menarche (14–16 years), compared to the reference group (11–13 years), showed a slightly significant trend for both CMI‐1 (AOR 1.65, 95% CI 0.95–2.96; *p* = 0.083†) and CMI‐2 (AOR 1.45, 95% CI 0.96–2.22; *p* = 0.081†). Age was significantly linked to CMI‐3 in the bivariate analysis (COR 1.23, 95% CI 1.04–1.48; *p* = 0.019), but this association was not maintained after multivariable adjustment (AOR 1.18; *p* = 0.240). Notably, academic background, academic year, marital status, family type, current or pre‐admission residence, religion, dysmenorrhea, menorrhagia, and high perceived stress were not independently associated with CMI irregularity at any threshold in the full‐cohort adjusted model (Table 2).

Multivariable models stratified by academic groups showed a vital effect modification in Table 3. Among non‐medical students, late menarche (14–16 years) was a significant predictor at multiple thresholds: compared to those with menarche at 11–13 years, students with late menarche were 2.71 times more likely to meet CMI‐1 criteria (AOR 2.71, 95% CI 1.22–6.50; *p* = 0.019), 1.93 times more likely for CMI‐2 (AOR 1.93, 95% CI 1.06–3.57; *p* = 0.034), and 2.37 times more likely for CMI‐3 (AOR 2.37, 95% CI 1.08–5.31; *p* = 0.033). This association was completely absent among medical students (all *p* > 0.8), indicating a substantial effect modification by academic background.

**Table 3:** Stratified multivariable analysis of factors associated with composite menstrual irregularity, by academic background.

Predictor variable	Model 1				Model 2				Model 3			
	CMI‐1: MI score ≥ 1				CMI‐2: MI score ≥ 2				CMI‐3: MI score ≥ 3			
	Non‐Medical		Medical		Non‐Medical		Medical		Non‐Medical		Medical	
	AOR (95% CI)	*p*‐value	AOR (95% CI)	*p*‐value	AOR (95% CI)	*p*‐value	AOR (95% CI)	*p*‐value	AOR (95% CI)	*p*‐value	AOR (95% CI)	*p*‐value
Academic year (ref: 1st year)												
2nd year	1.23 (0.42–3.55)	0.705	1.91 (0.65–6.24)	0.254	0.84 (0.39–1.82)	0.657	0.81 (0.31–2.07)	0.670	0.53 (0.19–1.46)	0.224	1.00 (0.19–4.31)	0.997
3rd year	1.18 (0.35–4.07)	0.789	1.48 (0.44–5.01)	0.526	0.68 (0.26–1.76)	0.434	1.42 (0.52–3.89)	0.496	0.36 (0.08–1.34)	0.146	2.66 (0.64–12.09)	0.188
4th year	0.85 (0.23–3.03)	0.808	1.81 (0.42–7.83)	0.422	0.76 (0.28–2.05)	0.593	0.85 (0.26–2.75)	0.782	0.97 (0.29–3.36)	0.955	2.44 (0.52–12.60)	0.270
Marital status (ref: single)												
Married	0.36 (0.12–1.08)	0.065†	1.64 (0.24–33.12)	0.666	0.24 (0.08–0.65)	0.006**	2.97 (0.67–16.41)	0.170	0.35 (0.08–1.19)	0.117	3.21 (0.67–14.73)	0.129
Family type (ref: nuclear)												
Joint	1.82 (0.77–4.68)	0.187	0.86 (0.37–2.15)	0.739	1.63 (0.86–3.14)	0.137	0.61 (0.28–1.28)	0.200	1.08 (0.46–2.44)	0.847	0.43 (0.11–1.28)	0.162
Residence (ref: with family)												
Hostel	1.48 (0.44–6.15)	0.556	3.76 (0.95–25.81)	0.101	0.49 (0.19–1.20)	0.125	2.73 (1.17–6.72)	0.023*	0.68 (0.18–2.10)	0.524	1.31 (0.38–3.89)	0.648
Pre‐admission (ref: urban)												
Rural	0.86 (0.33–2.42)	0.765	0.57 (0.18–2.01)	0.365	1.07 (0.51–2.26)	0.852	1.01 (0.39–2.64)	0.979	1.34 (0.54–3.21)	0.521	0.49 (0.07–2.05)	0.382
Religion (ref: Muslim)												
Hindu	0.24 (0.03–2.27)	0.167	2.12 (0.33–41.93)	0.504	0.14 (0.02–0.73)	0.032*	1.25 (0.32–4.75)	0.741	0.32 (0.01–2.25)	0.329	0.49 (0.02–3.23)	0.532
Income (ref: < 20,000 BDT)												
20,000–40,000 BDT	9.06 (1.70–57.58)	0.012*	6.37 (1.10–39.38)	0.039*	1.04 (0.33–3.19)	0.950	1.32 (0.30–6.23)	0.711	0.54 (0.14–2.10)	0.360	0.72 (0.12–6.13)	0.739
40,001–60,000 BDT	1.57 (0.40–5.67)	0.496	2.50 (0.56–10.43)	0.209	0.53 (0.18–1.54)	0.248	0.69 (0.17–2.96)	0.594	0.67 (0.19–2.45)	0.524	0.61 (0.12–4.70)	0.586
> 60,000 BDT	1.36 (0.34–5.10)	0.656	3.62 (0.75–16.48)	0.095†	0.48 (0.16–1.45)	0.198	0.78 (0.19–3.47)	0.728	0.44 (0.12–1.69)	0.218	0.41 (0.07–3.36)	0.347
Age (years, continuous)	0.96 (0.66–1.42)	0.847	1.08 (0.70–1.69)	0.718	1.17 (0.87–1.60)	0.301	1.12 (0.79–1.59)	0.516	1.21 (0.81–1.81)	0.351	1.02 (0.65–1.61)	0.923
Menarche (ref: 11–13 years)												
9–10 years	6.48 (0.94–138.31)	0.112	0.53 (0.14–2.28)	0.365	1.93 (0.52–7.50)	0.325	2.82 (0.85–10.26)	0.097†	2.30 (0.41–10.25)	0.298	1.57 (0.35–5.95)	0.526
14–16 years	2.71 (1.22–6.50)	0.019*	1.09 (0.48–2.60)	0.832	1.93 (1.06–3.57)	0.034*	1.18 (0.61–2.27)	0.611	2.37 (1.08–5.31)	0.033*	1.07 (0.42–2.57)	0.891
Stress (ref: Low)												
High	0.63 (0.25–1.47)	0.308	1.32 (0.61–2.79)	0.477	1.19 (0.61–2.35)	0.617	1.47 (0.79–2.80)	0.228	0.75 (0.33–1.76)	0.501	1.88 (0.78–4.98)	0.180
Dysmenorrhea (ref: No)												
Yes	0.86 (0.40–1.80)	0.684	0.97 (0.46–2.07)	0.945	0.94 (0.52–1.67)	0.827	1.06 (0.58–1.92)	0.856	1.35 (0.62–2.98)	0.443	1.13 (0.51–2.54)	0.761
Menorrhagia (ref: no)												
Yes	2.31 (0.87–7.01)	0.110	1.01 (0.38–2.92)	0.989	1.03 (0.50–2.11)	0.936	1.43 (0.64–3.20)	0.384	1.89 (0.77–4.54)	0.157	1.48 (0.54–3.82)	0.426
Metrorrhagia (ref: no)												
Yes	4.18 (0.76–78.53)	0.182	4.41 (0.79–83.50)	0.169	7.86 (2.55–30.33)	< 0.001***	1.68 (0.59–4.95)	0.334	4.60 (1.60–13.13)	0.004**	2.89 (0.83–9.52)	0.082†

Abbreviation: AOR, adjusted odds ratio.

*** *p* < 0.001.

** *p* < 0.01.

* *p* < 0.05.

^†^ *p* < 0.10.

Among non‐medical students, being married was associated with a significantly lower likelihood of CMI‐2 than among single students (AOR 0.24, 95% CI 0.08–0.65; *p* = 0.006), a protective association not observed among medical students (AOR 2.97; *p* = 0.170). Hindu religion was associated with an 86% decrease in the odds of CMI‐2 among non‐medical students (AOR 0.14, 95% CI 0.02–0.73; *p* = 0.032), although small sample sizes limit interpretation. A marginal trend was seen for married status and CMI‐1 among non‐medical students (AOR 0.36, 95% CI 0.12–1.08; *p* = 0.065†).

Among medical students, residing in a hostel was independently linked to 2.73 times higher odds of CMI‐2 compared to living with family (AOR 2.73, 95% CI 1.17–6.72; *p* = 0.023), with a marginal trend toward CMI‐1 (AOR 3.76, 95% CI 0.95–25.81; *p* = 0.101†). No similar effect was observed among non‐medical students. Being in a higher income bracket (20,000–40,000 BDT vs. less than 20,000 BDT) was significantly associated with CMI‐1 in both non‐medical (AOR 9.06, 95% CI 1.70–57.58; *p* = 0.012) and medical students (AOR 6.37, 95% CI 1.10–39.38; *p* = 0.039).

Metrorrhagia remained a key predictor within groups. Among non‐medical students, it was linked to 7.86 times higher odds of CMI‐2 (AOR 7.86, 95% CI 2.55–30.33; *p* < 0.001) and 4.60 times higher odds of CMI‐3 (AOR 4.60, 95% CI 1.60–13.13; *p* = 0.004). Among medical students, metrorrhagia showed marginal trends for CMI‐1 (AOR 4.41; *p* = 0.169†) and CMI‐3 (AOR 2.89; *p* = 0.082†), with estimates of similar size but wider confidence intervals, likely due to fewer events in this group (Table 3).

Table 4 shows the sources of menstrual‐related knowledge. The internet and mass media were the primary sources of menstrual information overall (52.5%), followed by interpersonal sources (29.0%) and school (23.5%). Medical students more often cited the internet (57.2% vs 47.9%), reading relevant materials (28.8% vs 20.4%), and personal experience (28.4% vs 24.2%), while non‐medical students more frequently reported school‐based sources (25.4% vs 21.4%). Formal seminars were rarely mentioned in either group (3.6% overall) (Table 4).

**Table 4:** Sources of menstrual‐related knowledge among medical and non‐medical students.

Source of menstrual‐related knowledge	Non‐medical (%)	Medical (%)	Total (%)
Internet/mass media	115 (47.92%)	131 (57.21%)	246 (52.45%)
School	61(25.42%)	49 (21.40%)	110 (23.45%)
Interpersonal	68 (28.33%)	68 (29.69%)	136 (29.00%)
Seminar	9 (3.75%)	8 (3.50%)	17 (3.62%)
Personal experience	58 (24.17%)	65 (28.38%)	123 (26.23%)
Reading related materials	49 (20.42%)	66 (28.82%)	115 (24.52%)

**Figure 2:**
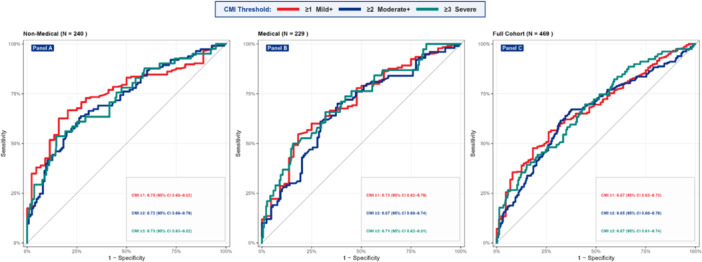
ROC of composite menstrual irregularity (CMI) at three severity thresholds (CMI‐1, CMI‐2, CMI‐3), stratified by academic background.

ROC analyzes showed moderate model performance across all CMI thresholds. For CMI‐1, the highest AUC was in non‐medical students (0.754, 95% CI 0.684–0.823), followed by medical students (0.705, 95% CI 0.622–0.789) and the whole cohort (0.674, 95% CI 0.617–0.731). At CMI‐2, AUCs were 0.721 (non‐medical), 0.675 (medical), and 0.652 (whole cohort); the non‐medical model had 73.0% specificity and balanced predictive values. At CMI‐3, the non‐medical model had an AUC of 0.725, 85.4% specificity, and 89.9% NPV, firmly ruling out severe irregularity, with moderate sensitivity (53.7%). Non‐medical models outperformed medical ones across thresholds (Figure 2; Supporting Information Table S2).

The multicollinearity analysis indicates that all multivariable models exhibited high diagnostic stability, with no significant multicollinearity detected among predictors. In the whole cohort, the Mean VIFs for the CMI‐1, CMI‐2, and CMI‐3 models were 1.696, 1.714, and 1.677, respectively (see Supporting Information Table S3). Additionally, stratified analyzes showed Mean VIFs ranging from 1.649 to 1.749 for non‐medical students and from 1.674 to 1.773 for medical students (see Supporting Information Table S4).

## 4 Discussion

This cross‐sectional study examined menstrual irregularity among 469 female undergraduate students in Chittagong, Bangladesh, using a novel three‐threshold Composite Menstrual Irregularity (CMI) framework. The CMI‐1, CMI‐2, and CMI‐3 prevalence estimates of 81.2%, 45.5%, and 16.9%, respectively, capture a clinically meaningful spectrum of severity. The CMI‐3 estimate of 16.9% is broadly consistent with global systematic review data reporting irregular menstruation in 5%–35.6% of reproductive‐aged women [6] while the CMI‐2 estimate (45.5%) aligns with South Asian studies reporting any menstrual disorder in 22%–48% of university students [45]. Subjective menstrual irregularity was significantly more prevalent among medical than non‐medical students (24.5% vs. 16.7%; *p* = 0.048), a distinction not reflected in CMI threshold distributions, suggesting a disconnect between subjective perception and composite multi‐parameter classification, a methodologically important finding that highlights the limitations of single‐question menstrual assessments.

Metrorrhagia was the strongest and most consistent independent predictor of composite menstrual irregularity across all three CMI thresholds (CMI‐1: AOR 4.37, 95% CI 1.26–27.65; CMI‐2: AOR 3.12, 95% CI 1.53–6.71; CMI‐3: AOR 3.27, 95% CI 1.53–6.83; all *p* ≤ 0.049). This robustness across severity levels identifies intermenstrual bleeding not merely as a co‐occurring symptom, but as a sentinel marker of underlying hormonal dysregulation, consistent with the FIGO PALM‐COEIN classification of abnormal uterine bleeding [46, 47]. Among non‐medical students, the association between metrorrhagia and irregularity was considerably stronger (CMI‐2: AOR 7.86; CMI‐3: AOR 4.60), possibly reflecting lower health literacy and delayed help‐seeking, resulting in more severe irregularity at the time of assessment. These findings call for integrating structured screening for intermenstrual bleeding into routine university health services.

Late menarche (14–16 years vs. 11–13 years) was a significant predictor of irregularity at all three CMI thresholds exclusively among non‐medical students (CMI‐1: AOR 2.71, *p* = 0.019; CMI‐2: AOR 1.93, *p* = 0.034; CMI‐3: AOR 2.37, *p* = 0.033), with no corresponding effect in medical students (all *p* > 0.8). This effect modification by academic background replicates findings from Korean and Polish university studies [48, 49] and is biologically explained by prolonged immaturity of the hypothalamic‐pituitary‐ovarian (HPO) axis in late‐maturing women. The absence of this association in medical students likely reflects earlier clinical recognition and management of menstrual symptoms facilitated by health sciences training. Among medical students, hostel residence was independently associated with moderate irregularity (CMI‐2: AOR 2.73, 95% CI 1.17–6.72; *p* = 0.023), implicating circadian disruption, sleep irregularity, and nutritional inconsistency inherent to dormitory living as modifiable determinants—findings consistent with evidence on HPO axis sensitivity to lifestyle disruption [20].

A paradoxical non‐linear income effect was observed: students in the 20,000–40,000 BDT income range showed markedly elevated CMI‐1 odds compared to the poorest group (AOR 5.52, 95% CI 1.78–17.88; *p* = 0.003), replicated in stratified models for both non‐medical (AOR 9.06) and medical students (AOR 6.37). This pattern is consistent with the nutritional transition hypothesis, which posits that lower‐middle‐income groups have sufficient economic access to energy‐dense, processed foods—promoting metabolic dysregulation—but lack the resources to access timely reproductive healthcare [45]. Although perceived stress (PSS‐10 > 20) was not independently associated with CMI outcomes in multivariable models—plausibly due to the ceiling effect of near‐universal high‐stress prevalence (71.2%), item‐level analysis revealed significant associations between stress reactivity and menstrual status (*p* = 0.012), consistent with findings from Japanese and South Asian student cohorts [4, 20]. The aggregate PSS threshold may be insufficiently sensitive to detect stress–cycle associations in samples with compressed stress distributions.

The internet and mass media were the primary sources of menstrual knowledge for 52.5% of participants, while formal seminars were cited by only 3.6%. This pattern mirrors national data indicating that almost half of Bangladeshi girls reach menarche without advance education [31], and reflects the systemic failure of reproductive health curricula across both medical and non‐medical higher education. AUC‐ROC values of 0.652–0.754 across models indicate moderate discriminatory performance, typical for self‐reported menstrual outcomes modeled from sociodemographic predictors, and are comparable to those reported in reference studies by Yamamoto et al. (2009) and Nohara et al. (2011) using similar frameworks [19, 20].

This study's primary strength is its multidimensional Composite Menstrual Irregularity (CMI) framework, which combines cycle length, duration, and flow volume to reduce misclassification bias. Methodological rigor is strengthened by a high response rate of 93.8%, stratified random sampling across medical and non‐medical faculties, and the use of three predefined CMI thresholds with AUC‐ROC analyzes. Additionally, including item‐level PSS‐10 analysis offers a more detailed view of the stress‐menstrual connection than previous regional studies literature.

Limitations include a cross‐sectional design and reliance on self‐reported data, which limit causal inference and may introduce recall bias. Additionally, the lack of clinical biomarkers, such as hormonal profiles or pelvic ultrasound, prevents distinguishing between functional and pathological irregularities. The study's focus on urban, young‐adult, predominantly Muslim university students in Chittagong may also limit generalizability to rural or older populations. Lastly, small sample sizes within certain demographic subgroups (e.g. married or Hindu participants) reduce the reliability of specific findings and sub‐analyzes.

## 5 Conclusion

To our knowledge, this is the first CMI‐based epidemiological analysis of menstrual irregularity among university students in Chattogram Bangladesh. This study highlights prevalence of menstrual irregularity and its associated factors, with over 60% of students reporting moderate to severe form of menstrual irregularity. The National Universities and Ministry of Health should require structured, evidence‐based menstrual health education in all undergraduate programs. Future research should emphasize longitudinal studies, biomarker‐enhanced assessments, nationally representative sampling, and intervention trials testing menstrual health education programs across Bangladesh's diverse female student population.

## Author Contributions

**Md. Mayin Uddin Hasan:** conceptualization, methodology, software, data curation, formal analysis, writing – original draft, visualization. **Brinta Bhowmik:** investigation, data curation, and writing – review and editing. **Mohammad Injamul Hoq:** writing – original draft, writing – review and editing, supervision, methodology. **Mohammed Aktar Sayeed:** resources, validation, and writing – review and editing. **Md. Zakir Sultan:** writing – review and editing, resources, supervision, validation.

## Funding

The authors have nothing to report.

## Ethics Statement

Ethical approval was obtained from the IRB of the Department of Public Health, University of Creative Technology, Chittagong, Chittagong‐4212, Bangladesh, for this work.

## Conflicts of Interest

The authors declare no conflicts of interest.

## Transparency Statement

The lead author Mohammad Injamul Hoq affirms that this manuscript is an honest, accurate, and transparent account of the study being reported; that no important aspects of the study have been omitted; and that any discrepancies from the study as planned (and, if relevant, registered) have been explained.

## Supporting information

Supporting File

## Data Availability

The data that support the findings of this study are available from the corresponding author upon reasonable request.
