# Supplementary material for: Composite Assessment of Menstrual Irregularity and Its Determinants Among Medical and Non‐Medical Female Undergraduates in Chittagong, Bangladesh: A Cross‐Sectional Study
Source: Health Sci Rep. 2026 Apr 27;9(5):e72448. doi: 10.1002/hsr2.72448 (PMC13121842; doi:10.1002/hsr2.72448)
Supplement: Supplementary file 1 — Supporting File [file HSR2-9-e72448-s001.docx]

**Supplementary Table 1:** PSS-10 Item-Level Analysis: Academic Background (Medical vs. Non-Medical) and MI (CMI ≥ 1).

| **PSS Item** | **Overall** | **Academic Background** | | | **Menstrual irregularity (CMI ≥ 1)** | | |
| --- | --- | --- | --- | --- | --- | --- | --- |
|  | Mean (SD) | Non-Medical Mean | Medical Mean | p Value | Regular Mean | Irregular Mean | P Value |
| Q1. ...been upset because of something that happened unexpectedly? | 2.33 (1.00) | 2.44 | 2.22 | **0.029^*^** | 2.07 | 2.39 | **0.012^*^** |
| Q2. ...felt that you were unable to control the important things in your life? | 2.20 (1.14) | 2.26 | 2.13 | 0.185 | 2.07 | 2.23 | 0.280 |
| Q3. ...felt nervous and "stressed"? | 2.50 (1.03) | 2.43 | 2.57 | 0.167 | 2.45 | 2.51 | 0.586 |
| Q4 (R). ...felt confident about your ability to handle your personal problems? | 2.48 (1.04) | 2.59 | 2.37 | **0.018^*^** | 2.47 | 2.48 | 0.910 |
| Q5 (R). ...felt that things were going your way? | 2.16 (0.87) | 2.17 | 2.14 | 0.744 | 2.12 | 2.16 | 0.691 |
| Q6. ...found that you could not cope with all the things that you had to do? | 1.96 (0.89) | 1.98 | 1.94 | 0.597 | 2.00 | 1.95 | 0.577 |
| Q7 (R). ...been able to control irritations in your life? | 2.25 (0.96) | 2.30 | 2.20 | 0.180 | 2.15 | 2.27 | 0.412 |
| Q8 (R). ...felt that you were on top of things? | 2.38 (0.97) | 2.39 | 2.38 | 0.533 | 2.35 | 2.39 | 0.964 |
| Q9. ...been angered because of things that were outside of your control? | 2.23 (1.05) | 2.29 | 2.17 | 0.357 | 2.08 | 2.27 | 0.155 |
| Q10. ...felt difficulties were piling up so high that you could not overcome them? | 1.98 (1.02) | 1.98 | 1.94 | 0.401 | 1.92 | 1.99 | 0.403 |

**Notes:**

- Scale: 0 (Never) to 4 (Very Often).
- (R): Indicates reverse-scored items.
- Statistical Test: Mann-Whitney U test used for all comparisons.
- Abbreviations: MNM (Academic Background: Medical vs. Non-Medical); MI (CMI ≥ 1).
- Statistical significance (p < 0.05).

**Supplementary Table 2: ROC Analysis Results by CMI Threshold and Study Group**

| **CMI Threshold** | **Metric** | **Non-Medical (n=239)** | **Medical (n=229)** | **Full Cohort (n=468)** |
| --- | --- | --- | --- | --- |
| **CMI ≥1** | AUC (95% CI) | 0.754 (0.684–0.823) | 0.705 (0.622–0.789) | 0.674 (0.617–0.731) |
|  | Sensitivity (%) | 62.6 | 54.6 | 55.8 |
|  | Specificity (%) | 84.1 | 81.8 | 73.9 |
|  | PPV (%) | 94.6 | 92.7 | 90.2 |
|  | NPV (%) | 33.6 | 30.0 | 27.9 |
|  | Accuracy (%) | 66.5 | 59.8 | 59.2 |
| **CMI ≥2** | AUC (95% CI) | 0.721 (0.656–0.786) | 0.675 (0.604–0.745) | 0.652 (0.602–0.703) |
|  | Sensitivity (%) | 63.7 | 70.0 | 67.1 |
|  | Specificity (%) | 73.0 | 62.0 | 62.4 |
|  | PPV (%) | 67.9 | 58.8 | 59.8 |
|  | NPV (%) | 69.2 | 72.7 | 69.4 |
|  | Accuracy (%) | 68.6 | 65.5 | 64.5 |
| **CMI ≥3** | AUC (95% CI) | 0.725 (0.634–0.816) | 0.713 (0.620–0.806) | 0.674 (0.608–0.740) |
|  | Sensitivity (%) | 53.7 | 50.0 | 69.6 |
|  | Specificity (%) | 85.4 | 84.3 | 55.5 |
|  | PPV (%) | 43.1 | 38.8 | 24.1 |
|  | NPV (%) | 89.9 | 89.4 | 90.0 |
|  | Accuracy (%) | 79.9 | 78.6 | 57.9 |

**Supplementary Table 3:** Variance Inflation Factor (VIF) Assessment — Full Cohort Multivariable Models (N = 468)

| **Predictor Variable** | **VIF CMI-1** | **VIF CMI-2** | **VIF CMI-3** |
| --- | --- | --- | --- |
| Academic Background (Medical vs Non-Medical) | 1.82 | 1.84 | 1.79 |
| Academic Year — 2nd Year | 2.11 | 2.13 | 2.09 |
| Academic Year — 3rd Year | 2.24 | 2.26 | 2.21 |
| Academic Year — 4th Year | 2.30 | 2.32 | 2.27 |
| Marital Status (Married vs Single) | 1.34 | 1.36 | 1.33 |
| Family Type (Joint vs Nuclear) | 1.29 | 1.31 | 1.28 |
| Current Residence (Hostel vs With Family) | 1.18 | 1.19 | 1.17 |
| Pre-Admission Residence (Rural vs Urban) | 1.22 | 1.23 | 1.20 |
| Religion (Hindu vs Muslim) | 1.09 | 1.10 | 1.08 |
| Family Income — 20,000–40,000 BDT | 2.43 | 2.45 | 2.41 |
| Family Income — 40,001–60,000 BDT | 2.61 | 2.63 | 2.58 |
| Family Income — >60,000 BDT | 2.57 | 2.59 | 2.54 |
| Age (years, continuous) | 1.47 | 1.49 | 1.45 |
| Age at Menarche — 9–10 years | 1.63 | 1.65 | 1.61 |
| Age at Menarche — 14–16 years | 1.71 | 1.73 | 1.69 |
| Perceived Stress (High vs Low) | 1.15 | 1.16 | 1.14 |
| Dysmenorrhea (Yes vs No) | 1.38 | 1.40 | 1.36 |
| Menorrhagia (Yes vs No) | 1.41 | 1.43 | 1.39 |
| Metrorrhagia (Yes vs No) | 1.28 | 1.29 | 1.27 |
| **Mean VIF (all predictors)** | **1.696** | **1.714** | **1.677** |

**Supplementary Table 4:** Variance Inflation Factor (VIF) Assessment — Stratified Models: Non-Medical (NM) vs. Medical (M)

| **Predictor Variable** | **Non-Medical Group (NM)** | | | **Medical Group (M)** | | |
| --- | --- | --- | --- | --- | --- | --- |
|  | **NM CMI-1 (≥1)** | **NM CMI-2 (≥2)** | **NM CMI-3 (≥3)** | **M CMI-1 (≥1)** | **M CMI-2 (≥2)** | **M CMI-3 (≥3)** |
| Academic Year — 2nd Year | 2.08 | 2.19 | 2.14 | 2.11 | 2.23 | 2.17 |
| Academic Year — 3rd Year | 2.20 | 2.33 | 2.26 | 2.23 | 2.36 | 2.29 |
| Academic Year — 4th Year | 2.25 | 2.38 | 2.31 | 2.28 | 2.41 | 2.34 |
| Marital Status (Married vs Single) | 1.30 | 1.42 | 1.35 | 1.32 | 1.44 | 1.37 |
| Family Type (Joint vs Nuclear) | 1.27 | 1.34 | 1.30 | 1.29 | 1.36 | 1.32 |
| Current Residence (Hostel vs With Family) | 1.16 | 1.21 | 1.18 | 1.18 | 1.22 | 1.20 |
| Pre-Admission Residence (Rural vs Urban) | 1.18 | 1.27 | 1.22 | 1.20 | 1.29 | 1.24 |
| Religion (Hindu vs Muslim) | 1.07 | 1.11 | 1.09 | 1.09 | 1.13 | 1.11 |
| Family Income — 20,000–40,000 BDT | 2.38 | 2.50 | 2.43 | 2.41 | 2.53 | 2.46 |
| Family Income — 40,001–60,000 BDT | 2.54 | 2.68 | 2.60 | 2.57 | 2.71 | 2.63 |
| Family Income — >60,000 BDT | 2.51 | 2.64 | 2.57 | 2.54 | 2.67 | 2.60 |
| Age (years, continuous) | 1.43 | 1.52 | 1.47 | 1.46 | 1.54 | 1.49 |
| Age at Menarche — 9–10 years | 1.58 | 1.70 | 1.63 | 1.61 | 1.73 | 1.66 |
| Age at Menarche — 14–16 years | 1.67 | 1.78 | 1.72 | 1.69 | 1.80 | 1.74 |
| Perceived Stress (High vs Low) | 1.12 | 1.18 | 1.15 | 1.14 | 1.20 | 1.17 |
| Dysmenorrhea (Yes vs No) | 1.34 | 1.44 | 1.38 | 1.36 | 1.46 | 1.40 |
| Menorrhagia (Yes vs No) | 1.37 | 1.47 | 1.41 | 1.39 | 1.49 | 1.43 |
| Metrorrhagia (Yes vs No) | 1.24 | 1.32 | 1.27 | 1.26 | 1.34 | 1.29 |
| **Mean VIF** | **1.649** | **1.749** | **1.693** | **1.674** | **1.773** | **1.717** |
